# Notoginsenoside R1 ameliorates diabetic encephalopathy by activating the Nrf2 pathway and inhibiting NLRP3 inflammasome activation

**DOI:** 10.18632/oncotarget.24295

**Published:** 2018-01-16

**Authors:** Yadong Zhai, Xiangbao Meng, Yun Luo, Yongmei Wu, Tianyuan Ye, Ping Zhou, Shilan Ding, Min Wang, Senbao Lu, Lili Zhu, Guibo Sun, Xiaobo Sun

**Affiliations:** ^1^ Beijing Key Laboratory of Innovative Drug Discovery of Traditional Chinese Medicine (Natural Medicine) and Translational Medicine, Institute of Medicinal Plant Development, Peking Union Medical College and Chinese Academy of Medical Sciences, Beijing, China; ^2^ Key Laboratory of Bioactive Substances and Resource Utilization of Chinese Herbal Medicine, Ministry of Education, Beijing, China; ^3^ Key Laboratory of Efficacy Evaluation of Chinese Medicine Against Glycolipid Metabolic Disorders, State Administration of Traditional Chinese Medicine, Beijing, China; ^4^ Department of Pharmacology, Changchun University of Traditional Chinese Medicine, Changchun, China; ^5^ College of Pharmacy, Harbin University of Commerce, Harbin, China; ^6^ Department of Bioengineering, Santa Clara University, Santa Clara, California, USA; ^7^ Guang’anmen Hospital, China Academy of Chinese Medical Sciences, Beijing, China

**Keywords:** Notoginsenoside R1, diabetic encephalopathy, oxidative stress, Nrf2 pathway, NLRP3 inflammasome

## Abstract

Numerous researches supported that oxidative stress and inflammation play important roles in the development of diabetic encephalopathy (DEP). Notoginsenoside R1 (NGR1), one major component of *Panax notoginseng*, is believed to have anti-oxidative, anti-inflammatory and neuroprotective properties. However, its neuroprotective effects against DEP and underlying mechanisms are still unknown. In this study, db/db mice as well as high-glucose (HG)-treated HT22 hippocampal neurons were used as *in vivo* and *in vitro* models to estimate NGR1 neuroprotection. NGR1 administration for 10 weeks could ameliorate cognitive dysfunction, depression-like behaviors, insulin resistance, hyperinsulinemia, dyslipidemia, and inflammation in db/db mice. NGR1 markedly decreased the oxidative stress induced by hyperglycemia in hippocampal neurons. NGR1 significantly activated the protein kinase B (Akt)/nuclear factor-erythroid 2-related factor2 (Nrf2) pathway, and inhibited NLRP3 inflammasome activation in hippocampal neurons, which might be essential for the neuroprotective effects of NGR1. Further supporting these results, we observed that pretreatment with the phosphatidylinositol 3-kinase inhibitor LY294002 abolished NGR1-mediated neuroprotective effects against oxidative stress and NLRP3 inflammasome activation in HG-treated HT22 hippocampal neurons. In conclusion, the present study demonstrates the neuroprotective effects of NGR1 on DEP by activating the Akt/Nrf2 pathway and inhibiting NLRP3 inflammasome activation. This study also provides a novel strategy for the application of NGR1 as a therapeutic agent for patients with DEP.

## INTRODUCTION

Type 2 diabetes mellitus (T2DM), characterized by hyperglycemia due to insulin resistance, impairs hippocampal structure and function. Recent epidemiological findings have indicated that diabetes mellitus (DM) is an independent risk factor of the development of cognitive dysfunction [1]. Patients with DM have a higher risk of developing Alzheimer’s disease (AD) and vascular dementia in the process of aging than non-DM control subjects [2, 3]. In addition, converging evidences have indentified that an augmented risk of neuropsychiatric disorders in DM [4, 5]. This complex complication of diabetes is recognized as diabetic encephalopathy (DEP) [6, 7], and its underlying mechanism is unclear. Impaired insulin signaling, advanced glycation end-product, neuronal apoptosis, vascular dysfunction, metabolic abnormalities, oxidative stress, endoplasmic reticulum stress, and inflammation were all involved in the development of DEP [7, 8].

Chronic metabolic inflammation in the hippocampus accelerates the development of neurodegenerative diseases. Interleukine-1β (IL-1β), an important pro-inflammatory cytokine, is involved in the development of diseases related to the central nervous system (CNS) [9]. The IL-1β maturation is mediated by the nucleotide binding and oligomerization domain-like (Nod) receptor family pyrin domain-containing 3 (NLRP3) inflammasome, which consists of a recognition receptor (NLRP3), an apoptosis-associated speck-like protein containing a card (ASC) and an effector molecule (caspase-1). The inhibition of NLRP3 inflammasome activation can ameliorate AD and neuropsychiatric disorders [10–12]. NLRP3 inflammasome is also implicated in diabetic complications. And inhibiting NLRP3 inflammasome activation can alleviate the complications of DM [13]. Moreover, hippocampal neurons treated with high glucose (HG) can activate NLRP3 inflammasome, which supported that NLRP3 inflammasome plays a vital role in the development of DEP [14].

Signaling intermediates that activate NLRP3 inflammasome remain unclear. Recent investigations showed that thioredoxin-interacting protein (TXNIP) is an important intermediate that binds to and activates NLRP3 in a mechanism dependent on reactive oxygen species (ROS) [15]. Heme oxygenase-1 (HO-1), an endogenous cytoprotective enzyme produced in response to oxidative stress, and its gene transcriptional activation are regulated by nuclear factor-erythroid 2-related factor2 (Nrf2) [16]. HO-1 exhibits potential properties of clearing ROS and anti-oxidative stress. Activating the hippocampal Nrf2/HO-1 pathway can improve learning and memory decline induced by obesity [17]. Nrf2 nuclear translocation is activated by the phosphatidylinositol 3-kinase /protein kinase B (PI3K/Akt) pathway [18]. Moreover, activating this pathway can lead to the reduction of TXNIP expression [19]. Therefore, activating the PI3K/Akt pathway and increasing HO-1 expres.+sion may provide a novel target to inhibit ROS/TXNIP/NLRP3 inflammasome.

**Figure 1:**
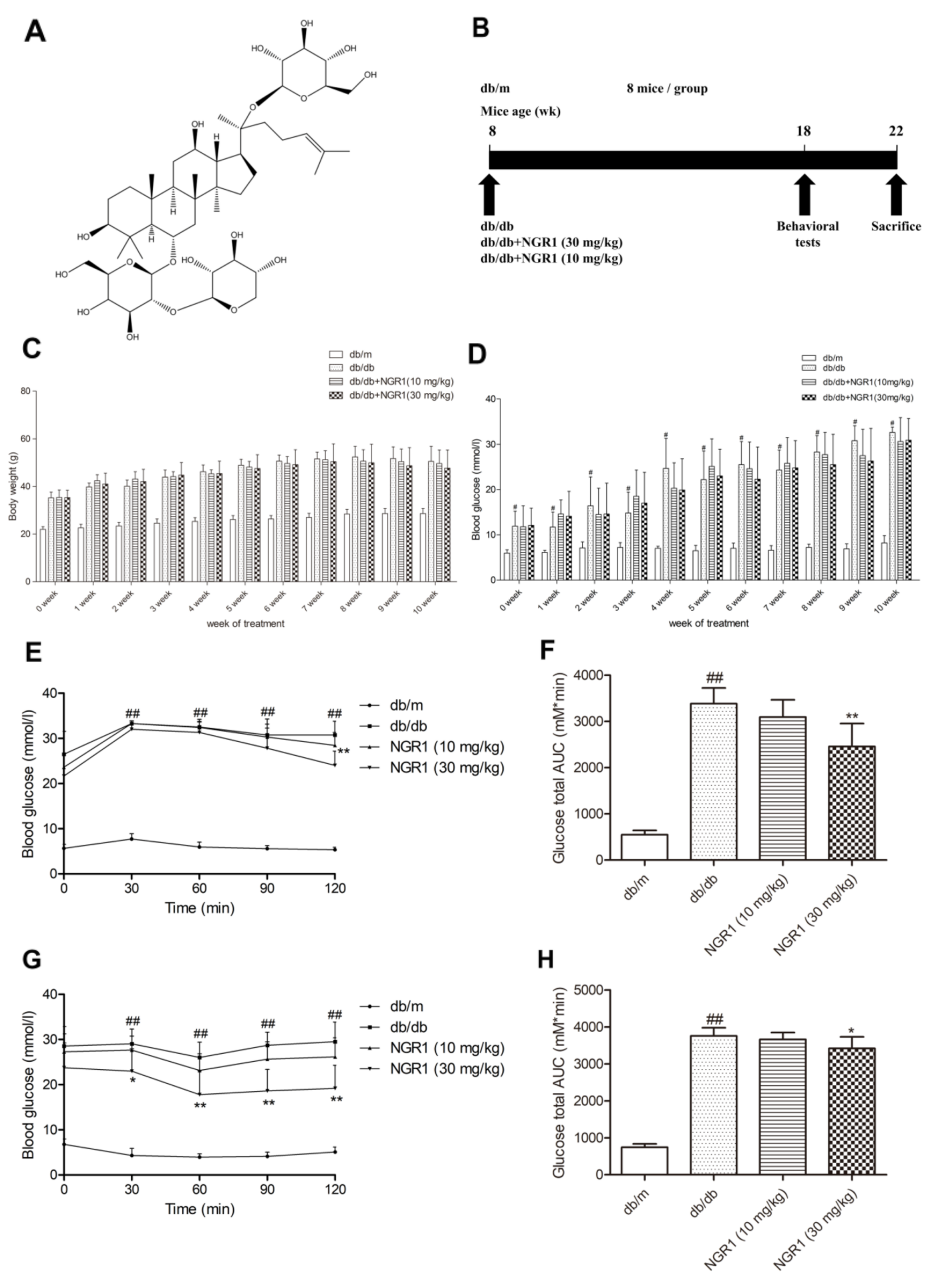
NGR1 improves insulin resistance in db/db mice **(A)** Chemical structure of NGR1; molecular weight is 933; molecular formula is C_47_H_80_O_18_. **(B)** Schematic diagram showing the timeline scheme of the animal experiments in *vivo*. **(C)** Body weights of mice in each group during 10 weeks of treatment. **(D)** Blood glucose level of mice in each group during 10 weeks of treatment. **(E)** Curve of blood glucose levels in OGTTs. **(F)** Glucose total AUC in OGTTs. **(G)** Curve of blood glucose levels in ITTs. **(H)** Glucose total AUC in ITTs. All data are represented as means ± SD for 8 mice in each group. ^##^ *P* < 0.01, compared with the db/m-group; ^**^ *p* < 0.01, ^*^ *p* < 0.05, compared with the db/db-group.

Saponins from *Panax notoginseng* can protect hippocampal neurons and improve spatial cognitive disorders in diabetic mice [20]. Notoginsenoside R1 (NGR1, its molecular structure is shown in Figure 1A), which is a major component and novel saponin isolated from *P. notoginseng*, exhibits anti-oxidative, anti-inflammatory, and anti-apoptotic properties [21]. In our previous study, NGR1 elicits neuroprotective effects against cerebral ischemia/reperfusion injury by activating the Akt/Nrf2/HO-1 pathway in *vivo* and in *vitro* [16]. NGR1 also attenuates Aβ_25–35_ induced injury in PC12 neuronal cells by suppressing oxidative stress, and inhibiting stress-activated mitogen-activated protein kinase (MAPK) signaling pathways [22]. Our previous research indentified that NGR1 exerts cardioprotective abilities against ischemia/reperfusion damage by inhibiting oxidative stress, endoplasmic reticulum stress, and cell apoptosis [23]. NGR1 also can improve the learning performance of APP/PS1 mice by increasing insulin degrading enzyme activity and inhibiting Aβ accumulation [24]. Moreover, NGR1 can ameliorate diabetic nephropathy in diabetic rats by activating PI3K/Akt signaling pathway, increasing nephrin and podocin expressions, decreasing desmin expression, and inhibiting inflammation and apoptosis of podocytes [25]. These studies emphasize the potential function of NGR1 in cerebrovascular diseases, neurodegenerative disorders, and diabetic complications. However, whether NGR1 can ameliorate DEP or whether its ameliorative effects are related to the inhibition of oxidative stress or NLRP3 inflammasome activation remains to be determined.

In this study, we investigated the neuroprotective effects and the underlying mechanisms of NGR1 on HG-induced HT22 hippocampal neurons injury, and oxidative stress [26, 27] and DEP in db/db mice, which display T2DM characteristic, including hyperglycemia, obesity, hyperinsulinemia, and insulin resistance and show neurobehavioral deficits, including cognitive dysfunction, depression, and anxiety [4, 5, 28, 29]. In *vivo* and in *vitro* analyses indicated that NGR1 could inhibit hyperglycemia induced oxidative stress and NLRP3 inflammasome activation through activating the Akt/Nrf2/HO-1 pathway in hippocampal neurons. Our findings demonstrated that NGR1 could be applied to treat and prevent DEP.

## RESULTS

### Effect of NGR1 on body weight and fasting blood glucose level in db/db mice

In Figure 1C and 1D, body weight and fasting blood glucose level significantly increased in the diabetic db/db mice compared with those in the non-diabetic db/m mice. No obvious differences in body weight and blood glucose level were found between the mice in the NGR1 (10 or 30 mg/kg) group and the mice in the model group until 10 weeks of treatment.

### NGR1 improved glucose tolerance and insulin sensitivity in db/db mice

As shown in Figure 1E, blood glucose level continuously increased at all time points during the oral glucose tolerance tests (OGTTs) in db/db mice compared with the db/m mice (*P*< 0.01). Glucose total area under the curve (AUC) of the model group was obviously increased compared with the control group (*P* < 0.01) (Figure 1F). Interestingly, administration of NGR1 (30 mg/kg) significantly decreased the blood glucose level at 120 min (*P* < 0.01) and greatly reduced the glucose total AUC compared with the model group (*P* < 0.05). In addition, treatment with NGR1 (30 mg/kg) showed an obvious difference in the rapid removal of blood glucose compared with the model group in insulin tolerance tests (ITTs) (*P* < 0.05, *P* < 0.01) (Figure 1G). As shown in Figure 1H, glucose total AUC of the NGR1 (30 mg/kg) group in ITTs was significantly decreased compared with the model group (*P* < 0.01).

### NGR1 attenuates depression-like behaviors and memory impairment in db/db mice

**Figure 2:**
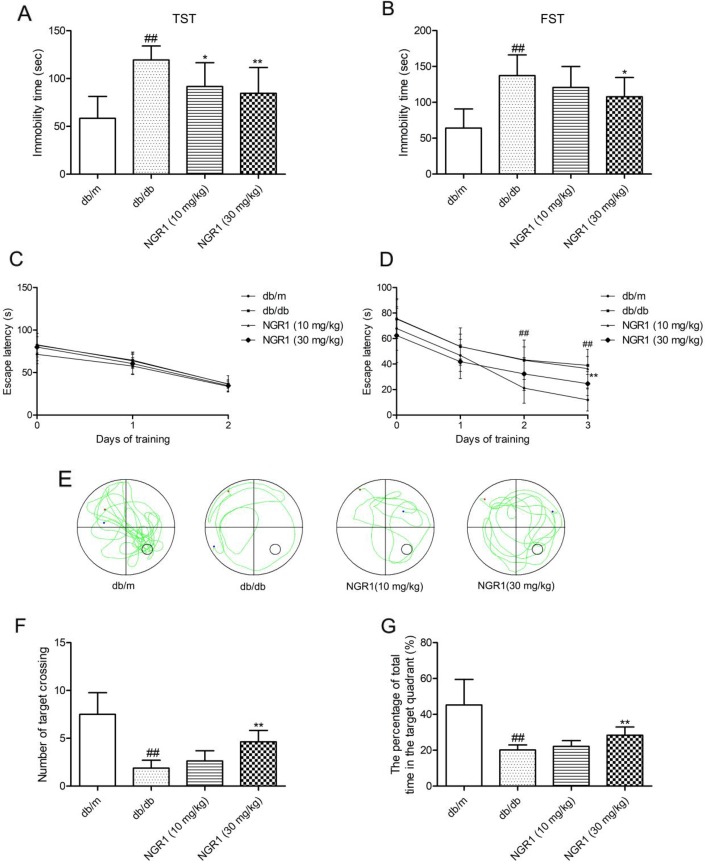
NGR1 attenuates depression-like behaviors and memory impairment in db/db mice In the Morris water maze (MWM) test, day 0 means performance on the first trial, and subsequent points indicate average of all daily trials. **(A)** Immobility time in TST. **(B)** Immobility time in FST. **(C)** Escape latency of the two-day visible-platform test. **(D)** Escape latency of the three-day hidden-platform test. **(E)** Representative swim paths during the probe test. **(F)** Percentage of total time spent in target quadrant in the probe trial. **(G)** Number of target crossings in the probe trial. Values are represented as means ± SD for 8 mice in each group. ^##^ *P* < 0.01, compared with the db/m-group; ^**^ *p* < 0.01, ^*^ *p* < 0.05, compared with the db/db-group.

Tail suspension test (TST) and forced swim test (FST) were regarded as classical experiments to evaluate depression. In the TST and FST, obvious differences were found among mice in the db/db and db/m groups. As shown in Figure 2A and 2B, db/db mice showed increased immobility time in TST and FST (*P* < 0.01), which were considered as more depressive than the wild type. Interestingly, NGR1 (10 and 30 mg/kg) treatment observably decreased the immobility time in TST and FST of db/db mice (*P* < 0.01). These data confirmed that NGR1 may improve depression-like behaviors in db/db mice.

As shown in Figure 2C, in the visible-platform test, the escape latency was similar in each group, which indicated no obvious differences in motivation and vision among all groups. In the spatial hidden-platform test (Figure 2D), the escape latency was shorter in the db/m group than in the model group in days 2 and 3 (*P* < 0.05). Interestingly, db/db mice treated with NGR1 (30 mg/kg) for 10 weeks showed remarkably reduced escape latency at day 3 compared with those in the model group. In the probe test (Figure 2E), a putative measurement of spatial learning and memory retention, db/db mice displayed less preference for the target quadrant. As shown in Figure 2F and 2G, the number of target crossings and the percentage of total time in the target quadrant were obviously decreased in db/db mice compared with those in db/m mice. By contrast, db/db mice treated with NGR1 (30 mg/kg) showed more preference for the target quadrant and higher frequency of crossing the platform compared with vehicle-treated db/db mice (*P*< 0.05). These data indicated that NGR1 may ameliorate memory disorder in db/db mice.

### NGR1 improves hyperinsulinemia and dyslipidemia in db/db mice

**Figure 3:**
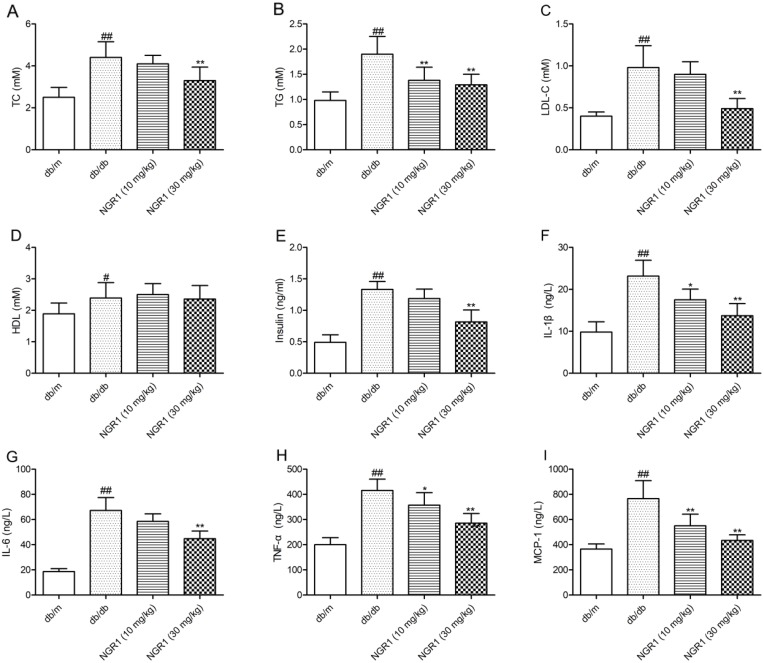
NGR1 influences lipids, insulin, and cytokines in plasma of db/db mice **(A)** Levels of TC in plasma samples of mice after 10 weeks of treatment. **(B)** Levels of TG in plasma samples of mice after 10 weeks of treatment. **(C)** Levels of LDL-C in plasma samples of mice after 10 weeks of treatment. **(D)** Levels of HDL-C in plasma samples of mice after 10 weeks of treatment. **(E)** Levels of insulin in plasma samples of mice after 10 weeks of treatment. **(F)** Levels of IL-1β in plasma samples of mice after 10 weeks of treatment. **(G)** Levels of IL-6 in plasma samples of mice after 10 weeks of treatment. **(H)** Levels of TNF-α in plasma samples of mice after 10 weeks of treatment. **(I)** Levels of MCP-1 in plasma samples of mice after 10 weeks of treatment. Values are represented as means ± SD for 8 mice in each group. ^##^ *P* < 0.01, ^#^ *P* < 0.05, compared with the db/m-group; ^**^ *P* < 0.01, ^*^ *P* < 0.05, compared with the db/db-group.

As shown in Figure 3A, 3B, and 3C, total cholesterol (TC), triglyceride (TG), and low-density lipoprotein cholesterol (LDL-C) levels in plasma were significantly increased in db/db mice compared with db/m mice; however, treatment with NGR1 (30 mg/kg) for 10 weeks observably decreased TC, TG, and LDL-C levels in db/db mice. As shown in Figure 3D, the level of high-density lipoprotein cholesterol (HDL-C) in plasma of db/db mice was higher than that in db/m mice. However, NGR1 treatment could not affect the HDL-C level in db/db mice. Moreover, as shown in Figure 3E, diabetic db/db mice showed a markedly higher level of plasma insulin than db/m mice. Interestingly, treatment with NGR1 (30 mg/kg) for 10 weeks exhibited effects in reducing plasma insulin levels in db/db mice. These data indicated that NGR1 can improve lipid and insulin disorders in plasma of db/db mice.

### NGR1 reduces increased plasma levels of inflammatory factors in db/db mice

The diabetic db/db mice showed higher plasma IL-1β, interleukine-6 (IL-6), tumor necrosis factor-α (TNF-α), and monocyte chemoattractant protein-1 (MCP-1) levels than db/m mice (*P* < 0.01) (Figure 3F, 3G, 3H, and 3I). Treatment with NGR1 (10 or 30 mg/kg) for 10 weeks lowered plasma IL-1β, TNF-α, and MCP-1 levels than vehicle-treated db/db mice (*P* < 0.01, *P* < 0.05). Moreover, NGR1 (30 mg/kg) treatment significantly reduced the plasma IL-6 level in db/db mice compared with those in the model group (*P* < 0.01).

### H&E, Nissl’s, and TUNEL staining

**Figure 4:**
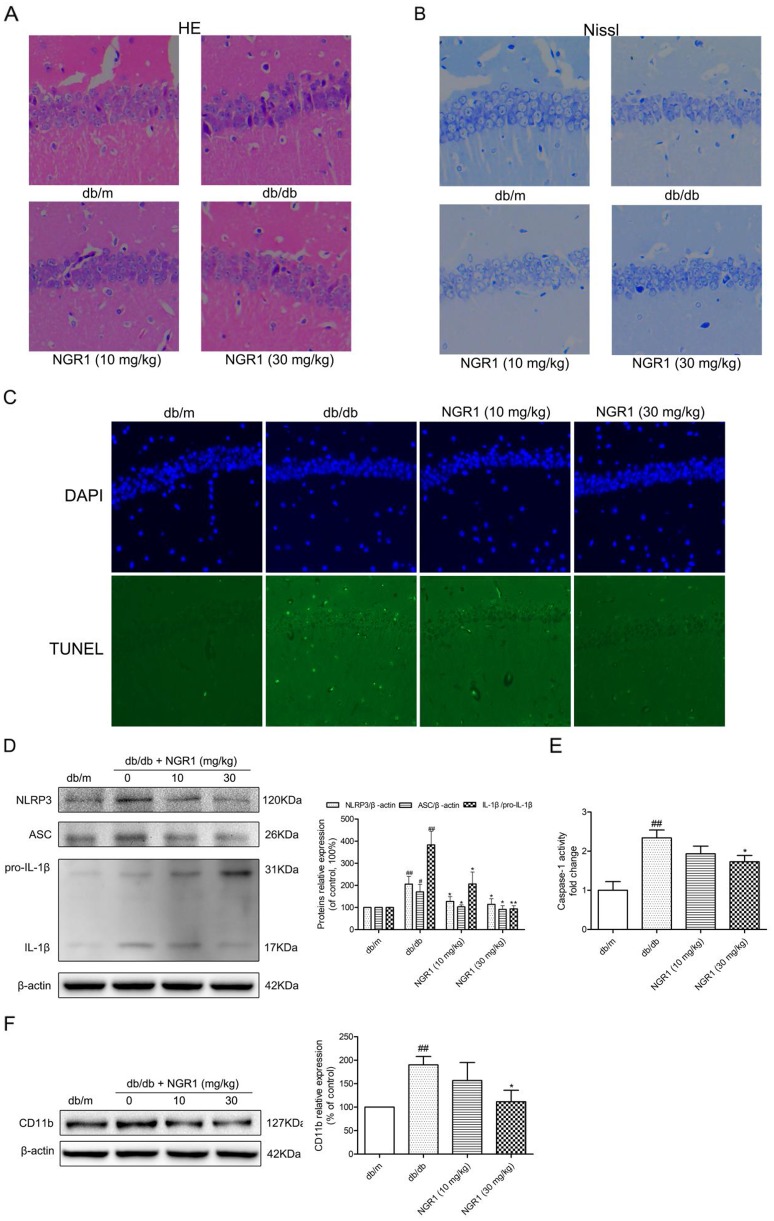
NGR1 exerts neuroprotective effects by inhibiting NLRP3 inflammasome activation in hippocampus of db/db mice **(A)** H&E staining in the hippocampal CA1 region for each group. **(B)** Nissl’s staining in the hippocampal CA1 region for each group. **(C)** TUNEL staining in the hippocampal CA1 region for each group. **(D)** Representative protein bands and Western blot analysis of NLRP3, ASC and IL-1β (P31 and P17) in the hippocampus of each group. **(E)** Caspase-1 activity in the hippocampus of each group. **(F)** Representative protein bands and Western blot analysis of CD11b in hippocampus of each group. All data are represented as means ± SD for 3 mice in each group. ^##^ *P* < 0.01, ^#^ *P* < 0.05, compared with the db/m-group; ^**^ *p* < 0.01, ^*^ *p* < 0.05, compared with the db/db-group.

As shown in Figure 4A, H&E staining showed the round and pale stained nuclei of neurons that were predominantly seen in the db/m group. In diabetic db/db mice, neurons showing pyknotic nuclei were seen in the CA1 regions of the hippocampus. Interestingly, administration of NGR1 (10 or 30 mg/kg) reduced the pyknotic nuclei in the CA1 regions of the hippocampus in db/db mice. As shown in Figure 4B, many neurons had shrunk phenotype and were irregularly scattered in the hippocampal CA1 region of db/db mice. Most neurons exhibited weak staining, which indicated that neurons were diffusely deteriorated and lots of Nissl bodies lost in these neurons. In contrast to the mice in db/db group, the mice in db/m group exhibited strong staining and possessed neurons arranged regularly in the hippocampal CA1 region. TUNEL staining was used to detect cell apoptosis. Figure 4C showed that there were many positive TUNEL cells in the hippocampal CA1 region of db/db mice, while there were almost no detectable positive TUNEL cells in db/m mice. NGR1 treatment decreased the cell apoptosis in the hippocampal CA1 region of db/db mice. The results of H&E, Nissl, and TUNEL staining indicated that NGR1 ameliorates the hippocampal damage in db/db mice.

### NGR1 inhibits hippocampal NLRP3 inflammasome activation in db/db mice

Compared with the db/m mice, the active IL-1β protein expression level was significantly increased in the hippocampus of db/db mice, which suggested activation of NLRP3 inflammasome in the hippocampus (Figure 4D). We detected caspase-1 activity (Figure 4E) and evaluated protein expression levels of NLRP3 and ASC in the hippocampus (Figure 4D). As expected, caspase-1 activity and NLRP3 and ASC expression levels in the hippocampus were significantly increased in db/db mice compared with db/m mice (*P* < 0.01). Interestingly, db/db mice treated with NGR1 (30 mg/kg) exhibited a significant decrease in caspase-1 activity (*P* < 0.05) as well as NLRP3, ASC, and IL-1β expression levels (*P* < 0.05) in the hippocampus compared with the db/db group. Administration of NGR1 could decrease NLRP3 inflammasome activation and IL-1β expression level to improve the hippocampal inflammation response in db/db mice.

Activated microglia cells are main source of central nervous system inflammatory cytokines [11, 30]. As described in Figure 4F, the expression microglia cell marker CD11b protein was significantly increased in the hippocampus of db/db mice as compared to db/m mice (*P* < 0.01). NGR1 (30 mg/kg) treatment significantly reduced hippocampal CD11b protein expression in db/db mice (*P* < 0.05), which suggested that NGR1 can ameliorate neuroinflammation.

### NGR1 reduces hippocampal oxidative stress by activating the Akt/Nrf2/HO-1 pathway in db/db mice

**Figure 5:**
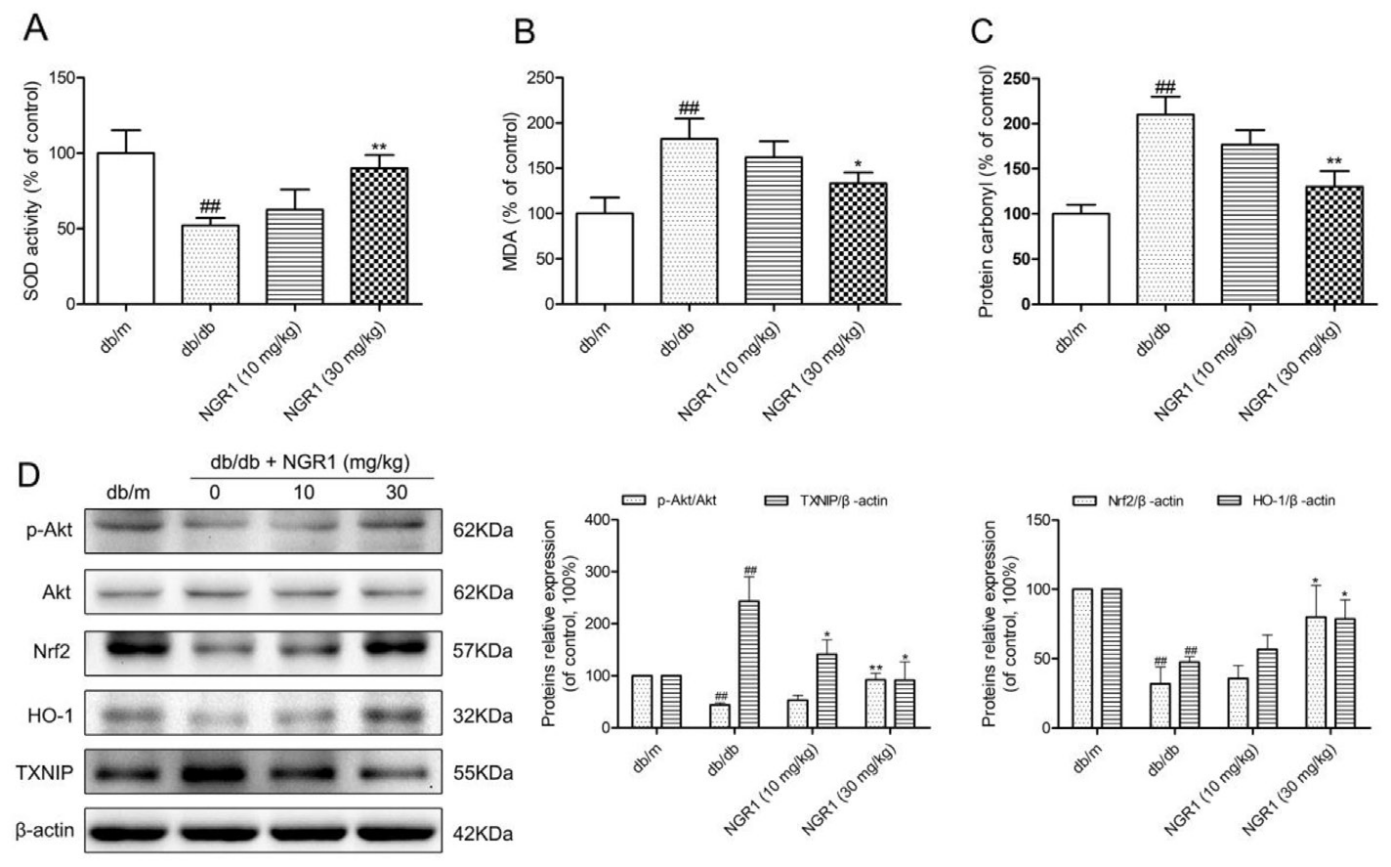
NGR1 inhibits oxidative stress by up-regulating the Akt/Nrf2/HO-1 pathway in the hippocampus of db/db mice **(A)** SOD activity in the hippocampus of each group. **(B)** MDA levels in the hippocampus of each group. **(C)** Protein carbonyl levels in the hippocampus of each group. **(D)** Representative protein bands and Western blot analysis of Akt, p-Akt, Nrf2, HO-1, and TXNIP in the hippocampus of each group. Values are represented as means ± SD for 3 mice in each group. ^##^ *P* < 0.01, compared with the db/m-group; ^**^ *P* < 0.01, ^*^ *P* < 0.05, compared with the db/db-group.

To determine whether oxidative stress was involved in hippocampal NLRP3 inflammasome activation in db/db mice, we detected hippocampal oxidative stress markers, including superoxide dismutase (SOD), malondialdehyde (MDA), and protein carbonyl. As shown in Figure 5A, 5B, and 5C, SOD activity significantly decreased, whereas MDA and protein carbonyl levels were remarkably increased in the hippocampus of db/db mice compared with the db/m group (*P* < 0.01). Moreover, NGR1 treatment could enhance anti-oxidant enzyme SOD activity and lower MDA, and protein carbonyl levels in the hippocampus of db/db mice (*P* < 0.05, *P* < 0.01). These results indicated that NGR1 treatment could suppress hippocampal oxidative stress in db/db mice, which may contribute to the inhibition of NLRP3 inflammasome activation.

Given that HO-1 exerts anti-inflammatory response and anti-oxidative stress properties, we examined hippocampal HO-1 expression. As shown in Figure 5D, db/db mice had lower HO-1 expression level than db/m mice (*P* < 0.01), which indicated that diabetes could suppress hippocampal HO-1 expression. It is well known that HO-1 expression is activated through nuclear transcription factor Nrf2, therefore, we further analyzed the hippocampal Nrf2 expression level. As expected, the Nrf2 expression level was lower in db/db mice than in db/m mice. Moreover, activation of the PI3K/Akt signal transduction pathway could promote Nrf2 nuclear translocation; thus, we detected the hippocampal Akt and phospho-Akt (p-Akt) expression. Similar to aprevious report [31], our study supported that expression levels of phosphorylated Akt were lower in diabetic db/db mice than in non-diabetic db/m mice (*P*< 0.01). This finding suggested that diabetes could also inhibit Akt phosphorylation in the hippocampus.

TXNIP, one of the important factors for NLRP3 inflammasome activation, can be activated by oxidative stress [15, 19]. As shown in Figure 5D, TXNIP expression levels were obviously increased in db/db mice (*P* < 0.01) compared with the db/m group. Interestingly, compared with the model group, treatment with NGR1 observably increased hippocampal phospho-Akt, Nrf2, and HO-1 expression levels and decreased TXNIP expression levels in db/db mice (*P* < 0.05). These results suggested that 10 weeks of NGR1 treatment could significantly activate the Akt/Nrf2 pathway and promote HO-1 expression, which result in a decrease in oxidative stress and NLRP3 inflammasome activation in the hippocampus of db/db mice.

### HG-induced HT22 hippocampal neurons injury, NLRP3 inflammasome activation and ROS production

**Figure 6:**
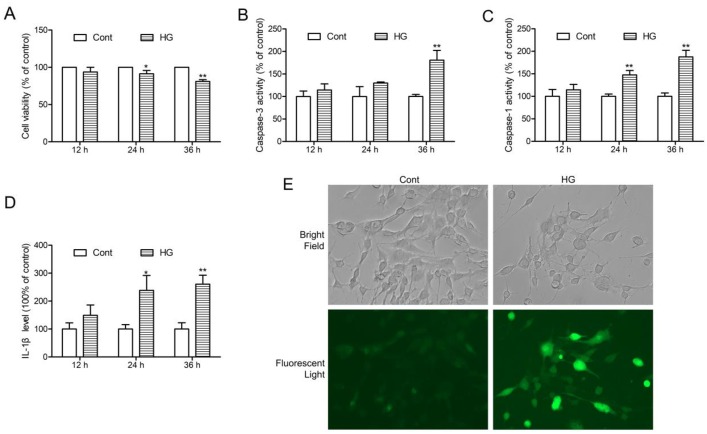
HG-induced injury, NLRP3 inflammasome activation and ROS production in HT22 hippocampal neurons **(A)** Followed by incubation with HG (50 mM vs control: 25 mM) at different time points (12, 24 and 36 h), and cell viability was detected by MTT assay. **(B)** Caspase-3 activity in HT22 hippocampal neurons after HG treatment at the indicated time periods. **(C)** Caspase-1 activity in HT22 hippocampal neurons after HG treatment at the indicated time periods. **(D)** IL-1β level in HT22 hippocampal neurons after HG treatment at the indicated time periods. **(E)** Intracellular ROS generation in HT22 hippocampal neurons after HG treatment for 36 h was visualised under a fluorescence microscopy. Values are represented as means ± SD from three independent experiments. ^**^ *P* < 0.01, ^*^ *P* < 0.05, compared with the control-group.

To investigate the roles of NGR1 in ameliorating DEP mechanistically, we established an *in vivo* model with HT22 hippocampal neurons exposed to HG (total 50 mM), as previously reported [26]. HT22 hippocampal neurons were exposed to HG for 12, 24, and 36 h. Cell viability were detected by MTT assay. The percentage of cell viability in each group was calculated relative to control. As shown in Figure 6A, 24 h HG treatment caused a decrease in cell viability approximately by 12%. Moreover, cell viability at 36 h after HG treatment was around 77%. We also detected caspase-3, and caspase-1 activities and IL-1β levels at 12, 24, and 36 h after incubation with HG. Interestingly, caspase-3, and caspase-1 activities and IL-1β level were higher at 36 h than at other time points (Figure 6B, 6C, and 6D). The intracellular ROS level was evaluated by detecting DCFH-DA fluorescence. As shown in Figure 6E, there was a significant increase in ROS production in HT22 hippocampal neurons at 36 h after HG treatment. On the basis of these results, incubation with HG for 36 h was selected as an optimal condition for the following experiments.

### NGR1 protects HT22 hippocampus neurons from hyperglycemia induced cell injury and intracellular oxidative stress

**Figure 7:**
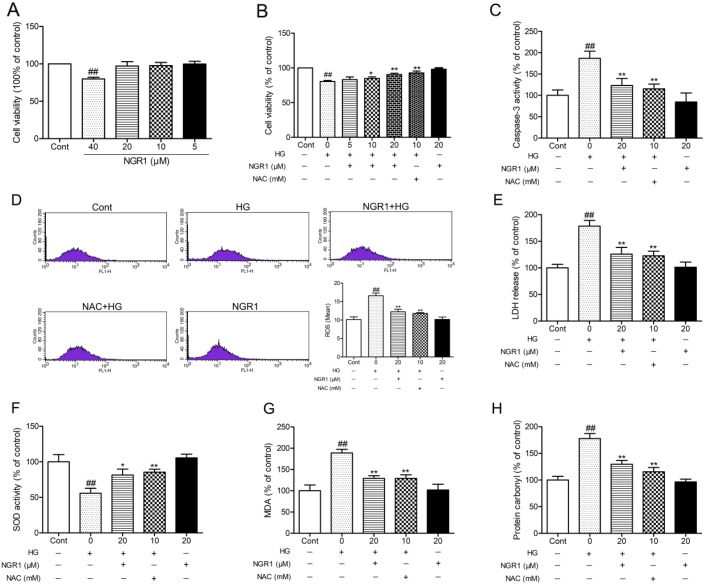
NGR1 ameliorates HG-induced cell injury, ROS production, and oxidative stress in HT22 hippocampal neurons **(A)** NGR1 showed no obvious cytotoxicity on cell viability with concentrations under 20 μM. **(B)** Effects of NGR1 on HG-induced cell viability were measured by MTT assay. **(C)** Effects of NGR1 on HG-induced intracellular caspase-3 activity. **(D)** Effects of NGR1 on HG-induced intracellular ROS levels detected using a FACSCalibur Fow Cytometer. **(E)** Effects of NGR1 on HG-induced LDH release. **(F)** Intracellular SOD activity in HG-induced HT22 hippocampal neurons. **(G)** Intracellular MDA levels in HG-induced HT22 hippocampal neurons. **(H)** Intracellular protein carbonyl levels in HG-induced HT22 hippocampal neurons. Values are represented as means ± SD from three independent experiments. ^##^ *P* < 0.01, compared with the control-group; ^**^ *P* < 0.01, ^*^ *P* < 0.05, compared with the model-group.

The potential neuroprotective effects of NGR1 on HT22 cells against HG-induced injury were evaluated by measuring cell viability, caspase-3 activity, and lactate dehydrogenase (LDH) release. Although high concentrations of NGR1 (40μM) showed certain cytotoxicity in HT22 cells that underwent incubation for 36 h, no obvious difference in cell viability was observed among the groups at low concentrations (5, 10, and 20 μM) and the control group (*P* > 0.05) (Figure 7A). N-acetyl-L-cysteine (NAC, 10 mM), a classical anti-oxidant, was used as a positive control [32]. As shown in Figure 7B, HT22 cells subjected to HG (50 mM) for 36 h showed significantly decreased cell viability (*P*< 0.01), and incubation with NGR1 obviously inhibited this decrease in a concentration-dependent manner (5, 10, and 20μM) (*P*< 0.05, *P*< 0.01). Moreover, HG treatment observably increased caspase-3 activity and LDH leakage in HT22 cells (*P* < 0.01) (Figure 7C, and 7E). And incubation with NGR1 (20 μM) observably decreased the caspase-3 activity and LDH leakage compared with the model group (*P* < 0.01). No obvious difference in cell viability, caspase-3 activity and LDH leakage was observed between the group treated with NGR1 (20 μM) alone and control group (*P* > 0.05). ROS is a mediator of glucose toxicity in HT22 neuronal cells [27]. The intracellular ROS level was detected by measuring carboxy-H2DCFDA fluorescence. As shown in Figure 7D, HG treatment observably increased intracellular ROS level in HT22 cells compared with the control group (*P* < 0.01). Compared with the model group, incubation with NGR1 (20 μM) obviously decreased intracellular ROS level (*P* < 0.01). These results showed that NGR1 treatment provides protection against HG-induced cell injury by inhibiting ROS production.

Intracellular oxidative stress was evaluated by measuring the content of lipid peroxidation product MDA, protein oxidation products protein carbonyl, and SOD activity. As shown in Figure 7F, 7G, and 7H, the SOD activity was observably decreased, whereas MDA, and protein carbonyl levels were observably decreased in HG-treated cells compared with the control group (*P* < 0.01). Interestingly, NGR1 (20 μM) treatment could enhance antioxidant enzyme SOD activity and lower MDA, protein carbonyl levels in HG-treated cells (*P* < 0.05). These results indicated that NGR1 can ameliorate HG-induced HT22 hippocampus neurons injury by inhibiting intracellular oxidative stress.

### NGR1 inhibits HG-induced NLRP3 inflammasome activation in HT22 hippocampal neurons

**Figure 8:**
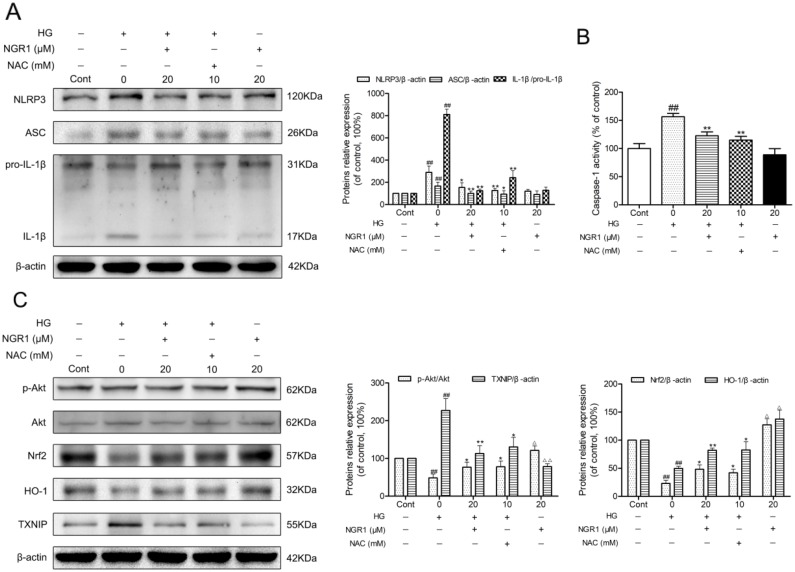
NGR1 activates the Akt/Nrf2/HO-1 pathway, and inhibits NLRP3 inflammasome activation in HG-induced HT22 hippocampal neurons **(A)** Representative protein bands and Western blot analysis of NLRP3, ASC, and IL-1β in hippocampal neurons. **(B)** Caspase-1 activity in hippocampal neurons. **(C)** Representative protein bands and Western blot analysis of Akt, p-Akt, Nrf2, HO-1, and TXNIP in hippocampal neurons. Values are represented as means ± SD from three independent experiments. ^##^ *P* < 0.01, ^△△^ *P* < 0.01, ^△^ *P* < 0.05 compared with the control-group; ^**^ *P* < 0.01, ^*^ *P* < 0.05, compared with the model-group.

Similar to previous studies, hyperglycemia caused NLRP3 inflammasome activation in HT22 hippocampal cells [14]. As shown in Figure 8A and 8B, NLRP3, ASC, and IL-1β expression levels and caspase-1 activity in HT22 cells were significantly elevated in the HG group compared with the control group (*P* < 0.01). Moreover, NGR1 (20 μM) treatment notably decreased NLRP3, ASC, and IL-1β expression levels and caspase-1 activity in HT22 cells compared with the non-treated HG group (*P* < 0.01).

To explore the molecular mechanism of NGR1 treatment on the inhibition of oxidative stress and NLRP3 inflammasome activation, Akt, p-Akt, nuclear Nrf2, HO-1, and TXNIP expressions were detected using Western blot analysis. As shown in Figure 8C, after 36 h exposure to hyperglycemia, phosphorylated Akt, nuclear Nrf2, and HO-1 expression levels were significantly decreased, whereas TXNIP expression level was remarkably increased compared with the control group (*P*< 0.01). Moreover, treatment with NGR1 (20 μM) obviously increased phosphorylated Akt, nuclear Nrf2, and HO-1 expression levels, while significantly decreased the TXNIP expression levels in the HT22 cells compared with the model group (*P* < 0.05, *P* < 0.01). Interestingly, a marked increase in phosphorylated Akt, unclear Nrf2, and HO-1 expression levels and a significant reduction in TXNIP expression levels were observed in HT22 cells treated with NGR1 alone (*P* < 0.05). These data indicated that NGR1 attenuates HG-induced injury in HT22 hippocampal neurons by activating the Akt/Nrf2/HO-1 pathway. Furthermore, NGR1 might promote the degradation of TXNIP by activating the PI3K/Akt pathway [19].

### PI3K inhibitor LY294002 abolishes the neuroprotective effects of NGR1 against HG-induced HT22 hippocampal neurons injury

**Figure 9:**
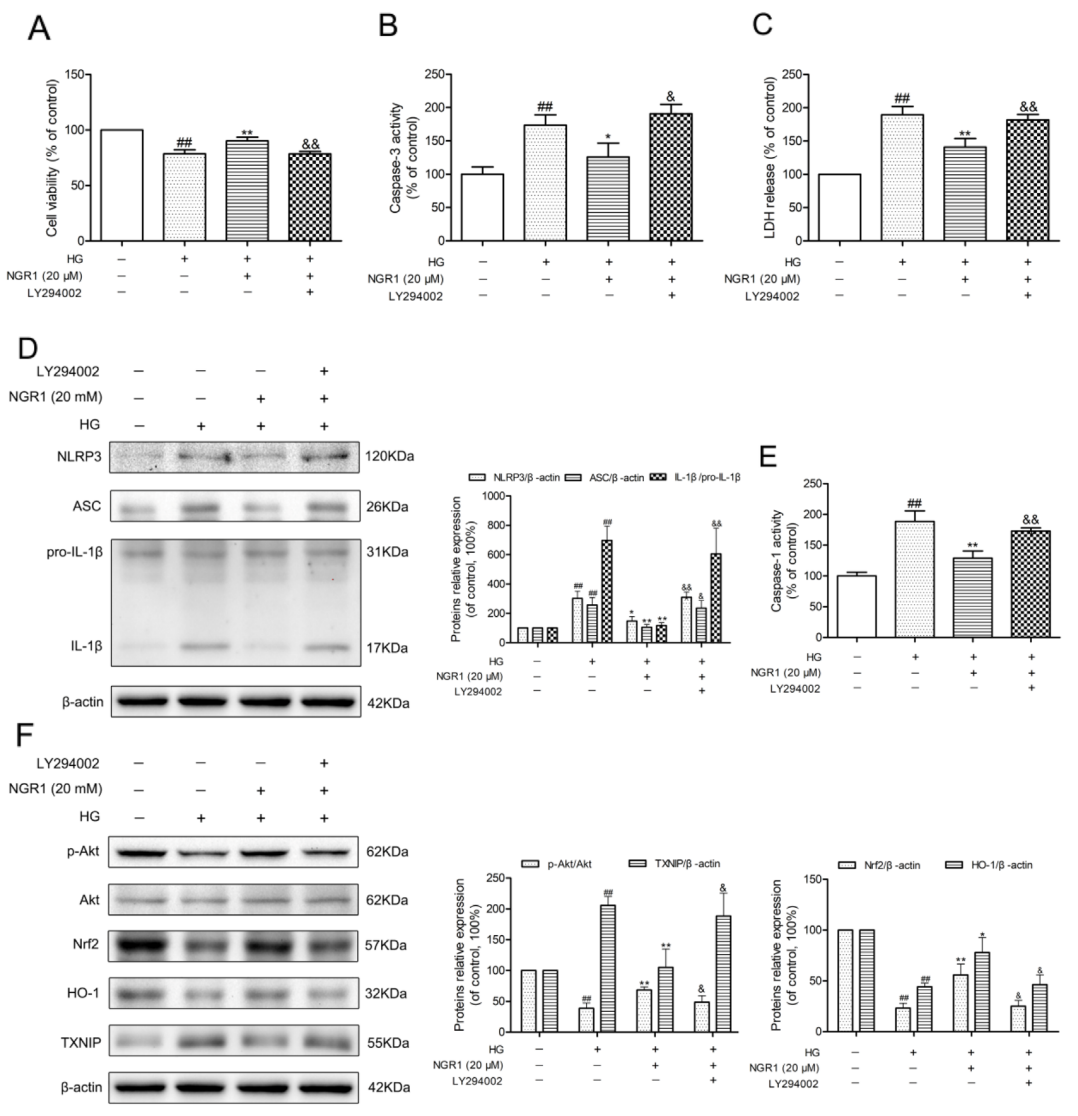
NGR1 exerts neuroprotective effects and inhibition of NLRP3 inflammasome by activating the Akt/Nrf2 pathway **(A)** Cell viability was measured by MTT assay. **(B)** Caspase-3 activity in HT22 hippocampal neurons. **(C)** LDH release in HT22 hippocampal neurons. **(D)** Representative protein bands and Western blot analysis of NLRP3, ASC, and IL-1β in hippocampal neurons. **(E)** Caspase-1 activity in hippocampal neurons. **(F)** Representative protein bands and Western blot analysis of Akt, p-Akt, Nrf2, HO-1, and TXNIP in hippocampal neurons. Values are represented as means ± SD from three independent experiments. ^##^ *P* < 0.01, compared with the control-group; ^**^ *P* < 0.01, ^*^ *P* < 0.05, compared with the model-group; ^&&^ *P* < 0.01, ^&^ *P* < 0.05, compared with the NGR1 (20 mM)-group.

To verify the effect of the Akt/Nrf2 pathway in the inhibition of oxidative stress and NLRP3 inflammasome activation in NGR1 treatment, the PI3K inhibitor LY294002 was used in experiments. As shown in Figure 9D, and 9E, the neuroprotective effect and inhibition of NLRP3 inflammasome activation of NGR1 were abolished by LY294002. Furthermore, LY294002 significantly decreased phosphorylated Akt, Nrf2 translocation, and HO-1 expression levels but TXNIP expression levels in HG- and NGR1-co-treated HT22 cells (*P* < 0.05) (Figure 9F). Most importantly, PI3K inhibitor LY294002 abolished the beneficial effect of NGR1 on the neuron injury in HG-induced HT22 cells (Figure 9A, 9B, and 9C). These results indicated that NGR1 exerts neuroprotective effects by activating the Akt/Nrf2/HO-1 pathway.

## DISCUSSION

In the present study, the diabetic db/db mice exhibited the behavioral characteristics of cognition impairment and depression, accompanied with hyperglycemia, excessive body weight, hyperinsulinemia, dyslipidemia, insulin resistance, and peripheral and central inflammation. Administration of NGR1 for 10 weeks could alleviate cognition decline, depression behaviors, and insulin resistance in db/db mice. And NGR1 treatment reduced peripheral inflammation and plasma TC, TG, LDL-C, and insulin levels in db/db mice. In addition, we also found that neuronal oxidative stress and NLRP3 inflammasome activation were involved in the ameliorative effects of NGR1 against DEP *in vivo* and *in vitro*. NGR1 reduced HG-induced oxidative stress by scavenging ROS, decreasing MDA, and oxidative carbonyl protein levels, increasing SOD activity and activating the Akt/Nrf2/HO-1 pathway. Moreover, our results identified that NGR1 is capable of decreasing TXNIP and NLRP3 inflammasome-related proteins expression both *in vivo* and *in vitro*. These findings suggested that inhibition of oxidative stress and NLRP3 inflammasome activation may be the vital mechanisms of NGR1 neuroprotection.

T2DM is a metabolic disease characterized by hyperglycemia, hyperinsulinemia, and dyslipidemia due to insulin resistance. Disordered lipid metabolism and hyperinsulinemia lead to numerous neurological diseases [33, 34]. Therefore, amelioration of hyperinsulinemia and dyslipidemia benefited the treatment of DEP. Similar to previous reports [35, 36], db/db mice exhibited high levels of TC, TG, LDL-C, and insulin in plasma. And administration of NGR1 for 10 weeks observably improved hyperinsulinemia and dyslipidemia in diabetic db/db mice.

Inflammation plays an important role in the onset of TD2M and progression of its complications. Numerous evidences suggested that patients with TD2M are under a state of subclinical chronic inflammation [8]. Excessive inflammation in T2DM can disturb the blood–brain barrier permeability, which allows access of toxic substances to the brain, contributing to the pathophysiological processes of many neurodegenerative diseases [37]. It is well known that activated microglial cells are main source of inflammatory cytokines in central nervous system and CD11b protein is a marker of microglial cells [11]. Our study showed that hippocampal CD11b was increased, suggesting that neuroinflammation occurred in db/db mice. Treatment with NGR1 could significantly decrease hippocampal CD11b expression in db/db mice. Moreover, administration of NGR1 could reduce IL-1β, IL-6, TNF-α, and MCP-1 levels in plasma of db/db mice. These data demonstrated that NGR1 ameliorates peripheral and central inflammation, which contributed to the improvement of DEP.

Researches in recent decades illuminated that IL-1β accelerates the pathogenesis of neurodegenerative diseases and diabetic complications [9, 38]. Hippocampal IL-1β expression level is observably increased, and this increase is related to cognitive and emotional alterations in diabetic mice [28]. The cleavage and maturation of IL-1β are activated by NLRP3 inflammasome, and the activation of NLRP3 inflammasome can contribute to pathophysiological processes involved in diabetic complications and neurodegenerative diseases [13, 39]. The knockdown of NLRP3 or caspase-1 in APP/PS1 mice can improve cognitive dysfunction [10]. Moreover, the inhibition of NLRP3 inflammasome activation can alleviate diabetic complications, including diabetic cardiomyopathy, diabetic nephropathy, diabetic retinopathy, diabetes-related wound-healing defects, and diabetic vascular endothelial dysfunction [40–44]. In the present study, high levels of IL-1β and NLRP3 inflammasome activation were observed in the hippocampus of the db/db mice and HG-induced hippocampal neurons, suggesting that hyperglycemia could activate NLRP3 inflammasome and induce hippocampal inflammation. Our data demonstrated that NGR1 could decrease NLRP3 inflammasome activation and IL-1β expression level *in vivo* and *in vitro*. Our results indicated that the inhibition of NLRP3 inflammasome is involved in the ameliorative effects of NGR1 against DEP.

The major upstream mechanisms of NLRP3 inflammasome activation include ROS, phagosomal destabilization, and ion fluxes [45]. Ample evidence showed that high rate of ROS is produced in hyperglycemia, thereby affecting neurons [27]. Our previous studies verified that NGR1 exerts neuroprotective and cardioprotective effects by inhibiting oxidative stress [16, 23]. Therefore, we focused on the ability of NGR1 to reduce oxidative stress by detecting the levels of oxidative stress markers (ROS, MDA, protein carbonyls, and SOD). In the present study, high level of ROS generation was observed in HG treated HT22 cells. And incubation with NGR1 could significantly reduce ROS production. Furthermore, our data showed that NGR1 significantly increases SOD activity and reduces MDA, and protein carbonyl levels in the hippocampus of db/db mice and HG-treated HT22 cells, which suggested its anti-oxidative efficiency.

TXNIP is a link between ROS and NLRP3 inflammasome activation [15]. ROS, a major upstream mechanism related to NLRP3 activation, induces the separation of TXNIP from thioredoxin and permits it to bind to NLRP3. TXNIP is a possible therapeutic target for diabetes and its related vascular complications [46]. In the present study, TXNIP expression level observably increased in the hippocampus of the diabetic mice and HG-treated HT22 cells. Moreover, treatment with NGR1 could remarkably decrease the expression of TXNIP *in vivo* and *in vitro*. Interestingly, there was a significant reduction in TXNIP expression level in HT22 cells incubated with NGR1 alone probably because of the activation of Akt phosphorylation [19].

HO-1 is an endogenous anti-oxidant protein that is activated by Nrf2-dependent signaling pathway. Previous studies have indicated that activating the PI3K/Akt pathway induces the translocation of Nrf2 to the nucleus and increases HO-1 expression in hippocampal neurons [47]. In addition, activating the Nrf2/HO-1 pathway can decrease NLRP3 inflammasome activation [17, 48, 49]. In our previous studies, NGR1 exhibited neuroprotective effects by activating the Akt/Nrf2 pathway and increasing HO-1 expression [16]. In the present study, higher expression levels of phosphorylated Akt, Nrf2, and HO-1 were observed in the HT22 cells treated with NGR1 alone than in the control group, indicating that NGR1 intervention activates the Akt/Nrf2 pathway and promotes HO-1 expression. Our data also indicated that the Akt/Nrf2/HO-1 pathway is inactivated in the hippocampus of db/db mice and HG-treated HT22 hippocampal neurons. NGR1 administration significantly increased the hippocampal phosphorylation of Akt, Nrf2, and HO-1 expression levels *in vivo* and *in vitro*. Interestingly, these changes were abolished by the PI3K inhibitor LY294002, thereby activating NLRP3 inflammasome and promoting TXNIP and IL-1β expression in hippocampal neurons. These results demonstrated that the neuroprotective properties of NGR1 were related to the up-regulation of the Akt/Nrf2/HO-1 pathway.

**Figure 10:**
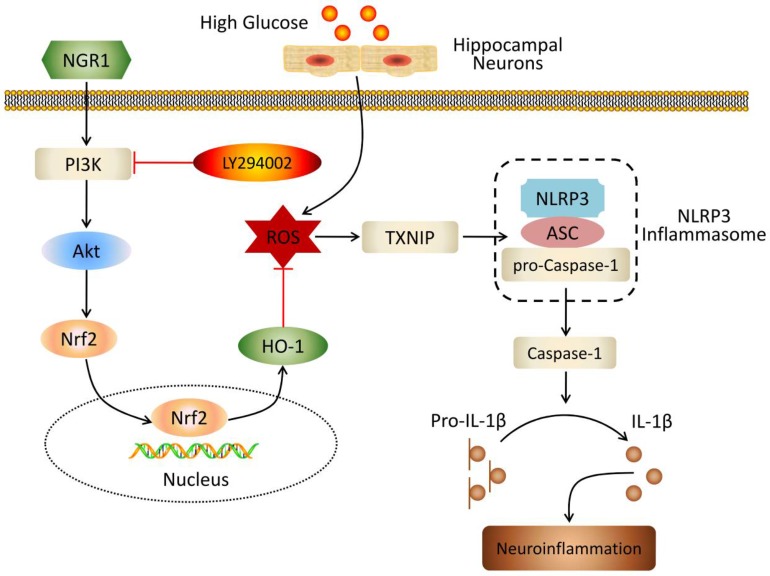
Schematic of NGR1 mechanism of ameliorating DEP by activating the Akt/Nrf2/HO-1 pathway and inhibiting NLRP3 inflammasome activation

In summary, our work demonstrated that NGR1 treatment significantly ameliorated DEP, and NGR1 improved insulin resistance and dyslipidemia in db/db mice. Our results indicated that NGR1 elicited neuroprotective effects by activating the Akt/Nrf2/HO-1 pathway, reducing oxidative stress, inhibiting NLRP3 inflammasome activation, and attenuating neuroinflammation (Figure 10). Therefore, these results showed that NGR1 may exhibit therapeutic properties for T2DM with DEP.

## MATERIALS AND METHODS

### Reagents and antibodies

Notoginsenoside R1 (NGR1, molecular weight = 933.14, CAS NO: 80418-24-2, purity>98.6) was obtained from Chengdu Must Biotechnology Co., Ltd (Chengdu, China). Dulbecco’s modifed Eagle medium (DMEM), penicillin/streptomycin, and foetal bovine serum (FBS), and were supplied by Gibco (NewYork, USA). 0.25% trypsin was purchased from Beijing Solarbio Science & Technology Co., Ltd. (Beijing, China). The ELISA kit for determining the mouse insulin level was obtained from ALPCO (Salem, USA). The ELISA kit for determining the protein carbonyl was obtained from Cell Biolabs (San Diego, USA). The ELISA kits for detecting the mouse IL-6 and TNF-α were purchased from DAKEWEI (Shenzhen, China). The ELISA kit for determining the mouse MCP-1 was obtained from Beijing Expandbiotech Co., Ltd. (Beijing, China). The ELISA kit for measuring IL-1β and reactive oxygen species assay kit, caspase-1 activity assay kit, caspase-3 activity assay kit, one step TUNEL apoptosis assay kit, proteinase K, 2-(4-Amidinophenyl)-6-indolecarbamidine (DAPI) dihydrochloride staining solution, and *N*-acetyl-L-cysteine (NAC) were purchased from Beyotime Institute of Biotechnology (Beijing, China). Carboxymethylcellulose sodium was bought from Amresco (Houston, USA). The kits for determining the lactate dehydrogenase (LDH), malondialdehyde (MDA), and superoxide dismutase (SOD) were obtained from Nanjing jiancheng Bioengineering Institute (Nanjing, China). The commercial kits for detecting TC, TG, LDL-C, and HDL-C were obtained from Biosino Biotechnology & Science Inc (Beijing, China). Carboxy-H2DCFDA was offered from Life Technologies (Carlsbad, USA). Primary antibodies against NLRP3, CD11b, TXNIP, and HO-1 were obtained from abcam (Cambridge, UK). Primary antibodies against Akt, ASC, IL-1β, and Nrf2 were purchased from Santa Cruz Biotechnology (Santa Cruz, CA, USA). Primary antibody against phospho-Akt (Ser473) was obtained from Cell Signaling Technology (Danvers, MA, USA). The peroxidase-conjugated secondary antibodies of goat anti-rabbit IgG and goat anti-mouse IgG were purchased from ZSJQ-BIO (Beijing, China). 3-(4,5-dimethylthiazol-2yl-)-2,5-diphenyl tetrazolium bromide (MTT), PI3K inhibitor LY294002, and all of other regents were obtained from Sigma-Aldrich (St. Louis, USA).

### Animals

Male 7-week old diabetic mice with a homozygous mutation of the leptin receptor (C57BLKS/J-lepr^db^/lepr^db^) and age-matched non-diabetic mice (db/m) were supplied by the Model Animal Research Center of Nanjing University (Nanjing, China). The animals were placed under temperature- and humidity-controlled laboratory conditions (temperature: 22 ± 2 °C, humidity: 60 ± 5 %). The mice were allowed free access to food and water, and were maintained under a 12 h light-dark cycle. All experimental procedures were approved by the Animal Ethics Committee of Peking Union Medical College.

### Experimental protocol

After 1 week of adaption, the diabetic db/db mice were randomly divided into three groups (n=8 each): model control group, NGR1 (30 mg/kg) group, and NGR1 (10 mg/kg) group. The non-diabetic db/m mice were as control group (n=8). The mice in control group and model group were administrated intragastrically with vehicle (0.5% carboxymethyl cellulose solution). NGR1 treated groups were administrated intragastrically with 30 mg/kg/day or 10 mg/kg/day NGR1 in vehicle. Drug administration was performed once daily at around 9 a.m. All mice were treated for 10 weeks. Body weight and fasting blood glucose level were detected every week. Blood glucose level was detected after 6 h of fasting food with a portable glucometer (Roche Group, Switzerland).

### Oral glucose tolerance test and Insulin tolerance test

Oral glucose tolerance test (OGTT) and insulin tolerance test (ITT) were performed as previous methods with slight modification [50]. After an overnight fast (12 h), OGTT or ITT were conducted by intragestrical administration of glucose solution (1 g/kg) or intraperitoneal injection of insulin (0.75 U/kg) in saline. Blood glucose level was detected at 0, 30, 60, 90, and 120 min after glucose administration or insulin injection, respectively.

### Behavioral tests

Experiments were performed at 9 a.m.-17 p.m. under conditions of dim light and low noise.

### Tail suspension test (TST)

The test was performed as described previously with slight modifications [5]. Each mouse was individually suspended by the tail to a vertical bar on the top of an opaque box (30 × 30 × 30 cm), with adhesive tape affixed 2 cm from the tip of the tail. A 6 min test was performed for each mouse. The immobility time was recorded for the last 4 min test. Immobility was set as the absence of any movements except those caused by respiration. The box was thoroughly cleaned using 75% alcohol before each use to remove odor cues.

### Forced swim test (FST)

The test was performed as previously described with little modifications [5]. Each mouse was placed into cylinder (20 cm height × 13 cm diameters) containing 25 °C water 15 cm deep so that the mouse could not support itself by contacting the bottom. A 6-min test for each mouse was videotaped by a camera placed above the cylinder. The immobility time was measured for the last 4 min. Immobility was defined as the absence of necessary movements except those required for respiration. The water temperature was controlled at 25 °C.

### Morris water maze test

The MWM test was performed according to the method described previously with minor modifications to assess spatial memory [51]. The test included a 5-day training (visible- and hidden- platform training sessions) and a probe trial on day 6. The water maze equipment included a circular pool (100 cm in diameter, 50 cm in height), a black platform (9 cm in diameter), and a computer equipped with a management system (Super Maze, Shanghai Xinruan Information Technology Co., Ltd. China). The mouse was trained in the pool filled up with water maintained at 25 ± 1 °C. The maze was located in a lit room with visual cues. The pool was spatially divided into four imaginary quadrants and the platform was placed in the center of one quadrant. The position of the platform was invariant during the visible-platform and hidden-platform training sessions. The visible-platform training was implemented to detect differences in the vision and motivation of each group. The platform was placed 1 cm below the water surface and marked with a small flag (5 cm in height) in the visible-platform training. The hidden-platform training was facilitated to evaluate the ability of spatial learning. The flag was removed and the platform was placed 1 cm underneath the water surface. For each trial per day, each mouse was performed to four trials with a 1 h interval. Escape latency data were recorded. The training trial began with placing the animal in the water facing the wall of the pool and drop location was randomly changed for each trial. Each trial lasted until the mouse reached the platform and stayed there for 10 s. If the mouse failed to find the platform within 90 s, the trial was ended and the mouse was guided to the platform for 30 s; its escape latency was recorded as 90 s. On day 6, the probe trial began. Each mouse was allowed to swim freely in the pool for 90 s without the platform. The time spent in the target quadrant and the numbers of mice crossing through the original platform position were recorded.

### Preparation of tissue samples

After completion of the behavioral tests, overnight fasted mice were anesthetized by isoflurane inhalation. Blood samples were collected by cardiac puncture into EDTA (10%)-coated chilled tubes. After centrifugation (10 min, 3000 g, 4 °C), plasma were stored at −80 °C for further measurement. Three mice from each group were transcardially perfused with PBS followed by a 4% paraformaldehyde fixative solution. Brains were gently removed, immersed for 24 h in fixative, and then processed for subsequent experiment. The remaining mice of each group were perfused with cold PBS through the ascending aorta. Mice were decapitated and prefrontal cortex and the hippocampus were rapidly and carefully dissected on ice plate. The tissues were immediately collected into labeled-sterile tubes and frozen in liquid nitrogen and then stored at −80 °C until assays.

The plasma from each mouse were used to detect cytokines (L-1β, IL-6, TNF-α, and MCP-1), insulin, and lipids (TC, TG, LDL-C, and HDL-C). Three brains in 4% paraformaldehyde fixative solution from each group were used for H&E, Nissl’s, and TUNEL staining. The remaining hippocampal tissues were used for biochemical detection and western blotting.

### Measurement of plasma cytokines, lipids, and insulin

The levels of plasma cytokines (IL-1β, IL-6, TNF-α, and MCP-1) and insulin were measured by enzyme-linked immune sorbent assay (ELISA) kits following the manufacturer’s instructions as previously described. The levels of TC, TG, LDL-C, and HDL-C were detected by a Hitachi7600 Automatic Biochemistry Analyzer (Tokyo, Japan) according to the manufacturer’s instructions.

### Measurement of caspase-1, caspase-3, SOD, MDA, and protein carbonyl

The activities of caspase-1, caspase-3, and SOD and contents of MDA, and protein carbonyl were detected by the kits following the manufacturer’s instructions.

### H&E and Nissl’s staining

The fixed brains were embedded in paraffin and coronally dissected into 5 μm thick sections. In order to assess the damage of hippocampus, the brain paraffin sections were processed as described previously for histopathological examination by H&E staining [7]. The brain paraffin sections were also conducted by the method as described previously for Nissl’s staining. Images were analyzed by using a light microscope (EVOS^®^ XL Core, Life Technologies).

### Terminal deoxynucleotidyl transferase-mediated dUTP-biotin nick end labeling (TUNEL) assay

Cell apoptosis was assessed by a one step TUNEL apoptosis assay kit according to the previously described method [52]. In brief, brain paraffin sections were dewaxed and rehydrated, and then incubated in proteinase K working solution at 25 °C for 30 minutes. After being washed in PBS, sections were treated with TUNEL reaction mixture for 1 h at 37 °C in dark. And then, sections were incubated with DAPI solution for 3 minutes. Images were captured using a fluorescence microscope (EVOS^®^ FL Color, Life Technologies).

### Western blot analysis

Western blot was performed as previous report [53]. The tissues of hippocampus were weighed and homogenized in lysis buffer (1: 100 inhibitor proteases and phosphatases cocktail). In contrast to hippocampal tissues, the HT22 cells were lysed in sample buffer and sonicated by an ultrasonic cell disrupter. Total protein was determined by bicinchoninic acid (BCA) kits. The primary antibodies NLRP3 (1:1000), ASC (1:200), IL-1β (1:200), TXNIP (1:1000), Akt (1:200), p-Akt (1:1000), CD11b (1:1000), Nrf2 (1:200), HO-1 (1:1000) and β-actin (1:1000) were used for blotting. The proteins were visualized using a super enhanced chemiluminescence reagent. Western blotting images were analyzed using Image Lab software (BIO-RAD, USA).

### Cell culture and treatment

The HT22 cell (mouse hippocampal neuronal cell line), which has served as a successful extracorporeal model in the study of diabetes associated hippocampal damage, were obtained from Beijing Beina Chuanglian Biotechnology Institute (Beijing, China). The cells were cultured in high glucose DMEM medium (25 mM glucose) containing 10% FBS, 100 U/ml penicillin, and 100 mg/ml streptomycin at 37 °C with 5% CO_2_ [26]. After reaching 80% confluency, the cells were trypsinized and processed for subsequent experiment. Cells treated with different concentrations of NGR1, were incubated with HG (DMEM containing additional 25 mM glucose, total 50 mM glucose; HG group) for 12, 24, 36 hours. Control group did not add any additional glucose (total 25 mM glucose; control group).

### Detection for cell viability and LDH release

The cell viability of HT22 cells was evaluated by MTT assay [26]. Cells were seeded in 96-well cell culture plate at the seeding density of 4 × 10^3^/well for 24 h. After incubation with glucose or drugs, culture medium was replaced with MTT medium, and further incubated at 37 °C for 4 h. 150μl of DMSO was added to each well with shaking 10 min, before reading the plate. The optical density (OD) value was detected by a microplate reader (Infinite M1000, Tecan Sunrise, Austria) at wavelength of 570 nm. The relative cell viability (%) was calculated by the following formula: (OD value of experimental group) / (OD value of control group) × 100%.

Cell death was evaluated by LDH release. The medium of the HT22 hippocampus neurons was collected to detect LDH release by kit. Data were presented as relative levels of the control group.

### Measurement of intracellular ROS production by fluorescence microscopy and flow cytometry

The intracellular ROS production was monitored using a fluorescent probe DCFH-DA. After treatment, cells were incubated with 10 μM DCFH-DA for 25 min at 37 °C and then washed thrice with phosphate-buffered saline (PBS) buffer. Finally, cellular morphology and fluorescence distributions were observed using a fluorescence microscopy (EVOS^®^ FL Color, Life Technologies).

After treatment, cells were harvested by 0.25% trypsin and washed by PBS buffer. And then cells were centrifuged and incubated with 5-(and-6)-carboxy-2′, 7′-dichlorodihydrofluorescein diacetate (carboxy-H2DCFDA) in the dark at 37 °C for 30 min. The fluorescence was analyzed by a flow cytometry (BD, Biosciences, CA, USA).

### Statistical analysis

All data were expressed as means ± standard deviation (SD). The results were analyzed by a one way analysis of variance (ANOVA) based on Student’s two-tailed unpaired *t*-test. The *P* values less than 0.05 were considered to be statistically significant.

## Abbreviations

AD: Alzheimer’s disease
ANOVA: a one way analysis of variance
BCA: bicinchoninic acid
CNS: central nervous system
DAPI: 2-(4-Amidinophenyl)-6-indolecarbamidine
DEP: diabetic encephalopathy
DM: diabetes mellitus
DMEM: Dulbecco’s modifed Eagle medium
FBS: foetal bovine serum
FST: forced swim test
HG: high glucose
HDL-C: high-density lipoprotein cholesterol
HO-1: Heme oxygenase-1
IL-1β: Interleukine-1β
IL-6: Interleukine-6
ITT: insulin tolerance test
LDH: lactate dehydrogenase
LDL-C: low-density lipoprotein cholesterol
MAPK: mitogen-activated protein kinase
MCP-1: monocyte chemoattractant protein-1
MDA: malondialdehyde
MWM: Morris water maze
MTT: 3-(4
NAC: *N*-acetyl-L-cysteine
NGR1: Notoginsenoside R1
Nrf2: nuclear factor-erythroid 2-related factor2
OD: optical density
OGTT: oral glucose tolerance test
PI3K: phosphatidyl inositol 3-kinase
ROS: reactive oxygen species
SD: standard deviation
SOD: superoxide dismutase
TC: total cholesterol
T2DM: type 2 diabetes mellitus
TG: triglyceride
TNF-α: tumor necrosis factor-α
TST: tail suspension test
TUNEL: Terminal deoxynucleotidyl transferase-mediated dUTP-biotin nick end labeling
TXNIP: thioredoxin-interacting protein;
